# PKD3 promotes metastasis and growth of oral squamous cell carcinoma through positive feedback regulation with PD-L1 and activation of ERK-STAT1/3-EMT signalling

**DOI:** 10.1038/s41368-021-00112-w

**Published:** 2021-03-10

**Authors:** Bomiao Cui, Jiao Chen, Min Luo, Yiying Liu, Hongli Chen, Die Lü, Liwei Wang, Yingzhu Kang, Yun Feng, Libin Huang, Ping Zhang

**Affiliations:** 1 State Key Laboratory of Oral Diseases & National Clinical Research Center for Oral Diseases & West China Hospital of Stomatology, Sichuan University, Chengdu, China; 2 Department of Gastroenterology, West China Hospital, Sichuan University, Chengdu, China

**Keywords:** Head and neck cancer, Oral cancer

## Abstract

Oral squamous cell carcinoma (OSCC) has a high incidence of metastasis. Tumour immunotherapy targeting PD-L1 or PD-1 has been revolutionary; however, only a few patients with OSCC respond to this treatment. Therefore, it is essential to gain insights into the molecular mechanisms underlying the growth and metastasis of OSCC. In this study, we analysed the expression levels of protein kinase D3 (PKD3) and PD-L1 and their correlation with the expression of mesenchymal and epithelial markers. We found that the expression of PKD3 and PD-L1 in OSCC cells and tissues was significantly increased, which correlated positively with that of mesenchymal markers but negatively with that of epithelial markers. Silencing PKD3 significantly inhibited the growth, metastasis and invasion of OSCC cells, while its overexpression promoted these processes. Our further analyses revealed that there was positive feedback regulation between PKD3 and PD-L1, which could drive EMT of OSCC cells via the ERK/STAT1/3 pathway, thereby promoting tumour growth and metastasis. Furthermore, silencing PKD3 significantly inhibited the expression of PD-L1, and lymph node metastasis of OSCC was investigated with a mouse footpad xenograft model. Thus, our findings provide a theoretical basis for targeting PKD3 as an alternative method to block EMT for regulating PD-L1 expression and inhibiting OSCC growth and metastasis.

## Introduction

Head and neck squamous cell carcinoma (HNSCC) is the most common malignant tumour of the head and neck. Oral squamous cell carcinoma (OSCC), the most prevalent subtype of HNSCC, is characterised by aggressive invasion, early cervical lymph node metastasis, a high metastasis rate and fast growth.^1,2^ Treatment of patients with metastatic OSCC remains a major clinical challenge. Despite recent breakthroughs in tumour therapy research, the 5-year survival rate of patients with OSCC remains ~50%.^3^ Many tumour patients currently receive immunotherapy, which has been approved for the treatment of recurrent or metastatic HNSCC. Notwithstanding some success, only a few patients respond to this treatment. Therefore, understanding the mechanisms underlying the growth and metastasis of OSCC is crucial to develop new treatment strategies.

Epithelial–mesenchymal transition (EMT) is one of the critical processes that promotes metastasis.^4,5^ In the course of EMT, epithelial cells lose polarity, reorganise the actin cytoskeleton and acquire more mesenchymal characteristics, all of which enhance the cells’ metastatic ability. EMT is characterised by reduced expression of adherens junction proteins (such as E-cadherin) and increased expression of mesenchymal markers (such as N-cadherin, Snail and Vimentin), which occur via transcriptional and post-transcriptional mechanisms.^5–7^ Moreover, Snail can regulate the transcriptional repression of the epithelial marker E-cadherin (CDH1) during EMT.^8^ Hence, identifying potential EMT blockers in patients with OSCC may pave the way for promising new therapies.

Protein kinase D (PKD) is a member of a class of serine/threonine protein kinases that modulate numerous biological activities, such as protein transport, cell migration, differentiation, proliferation, apoptosis, EMT and immune regulation.^9–15^ PKD3 expression is increased in highly invasive breast and prostate cancers.^9–15^ The concomitant absence of PKD1 in highly invasive breast cancer indicates that while this protein plays an anti-tumour role, PKD3 may instead promote cancer. Unlike PKD1 and PKD3, PKD2 shows relatively unchanged expression during breast cancer progression.^13,15,16^ Moreover, downregulation of PKD3 expression has a greater effect on cell migration than that of PKD2 expression.^12^ Our research group obtained similar results in OSCC and found a significant correlation between the nuclear localisation of PKD3 and advanced tumour grade.^14^ Increasing evidence shows that PKD3 is involved in the signalling pathways of multiple oncogenes, including extracellular signal-regulated kinase 1/2 (ERK1/2), protein kinase B (AKT), nuclear factor-kappa B (NF-κB), and signal transducer and activator of transcription 1/3 (STAT1/3).^12,13,17^ These pathways can regulate EMT as well as tumour cell growth and metastasis.^18–22^ EMT may also contribute to the immune escape of tumour cells.^23^ ERK1/2, NF-κB and STAT1/3 are also important regulators of PD-L1 expression.^24^

Previously, we showed that PKD3 can regulate the expression of PD-L1. In this study, we investigated whether PKD3 participates in EMT of OSCC and examined the contribution of PD-L1 and related signalling pathways to the induction of the EMT phenotype.

## Results

### PKD3 is overexpressed in OSCC and is closely related to EMT

**Fig. 1:**
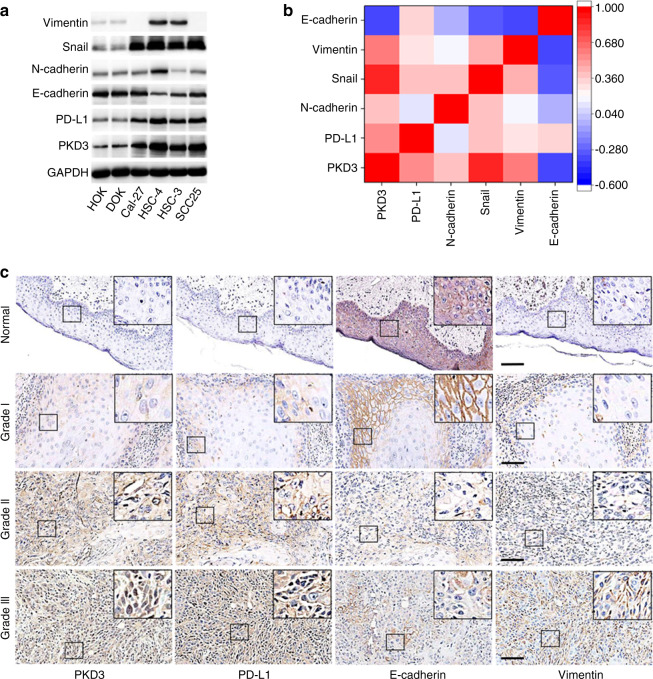
Expression of PKD3 in OSCC and its relationship with EMT. **a, b** The expression of PKD3, PD-L1 and EMT markers was detected by western blot in OSCC cells and non-tumour cells of the oral epithelium. Densitometry was analysed, and the correlation between PKD3, PD-L1, and EMT marker proteins was determined. **c** Expression of PKD3, PD-L1, and EMT markers in OSCC tissues detected by immunohistochemistry. Black scale bars are 100 µm. PKD3, protein kinase D3; OSCC, oral squamous cell carcinoma; PD-L1, programmed death ligand-1; EMT, epithelial-mesenchymal transition

Our previous studies determined that PKD3 can regulate PD-L1 expression.^14^ Moreover, PD-L1 is closely related to EMT in HNSCC.^25^ To verify whether PKD3 is involved in the regulation of EMT in OSCC, we first analysed the protein levels of PKD3, PD-L1 and EMT markers in OSCC cell lines and tissues using western blot and IHC. As shown in Fig. 1a, b, PKD3 was not only highly expressed in OSCC cell lines but also positively correlated with mesenchymal markers and negatively correlated with the epithelial marker E-cadherin. Moreover, the levels of PKD3, PD-L1 and Vimentin were higher in OSCC tissues than in normal tissues. With advanced tumour grade, the nuclear distribution of PKD3 as well as the levels of PKD3 and Vimentin gradually increased (Figs. 1c and S1a). However, the expression of PKD3 did not correlate with age, sex or race (Fig. S1b). Subsequently, we analysed the expression levels of PKD3 and PD-L1 in normal and HNSCC tissues using the UALCAN database. As shown in Fig. S1c, the levels of PKD3 and PD-L1 were significantly increased in tumour tissues compared to non-tumour tissues. We downloaded the RNA-seq data of the query genes from the UCSC Xena database and obtained the gene expression data of 564 HNSCC specimens to analyse the correlation of PKD3, PD-L1, and EMT-related markers. In this large dataset, the expression of PKD3 and PD-L1 was positively correlated with that of mesenchymal markers (such as SNAI1, SNAI2, ZEB1, ZEB2 and VIM) and negatively correlated with that of epithelial markers (such as CDH1, CLDN4 and CLDN7; Fig. S1d). In summary, our data indicate that PKD3 may play an important role in the occurrence and development of OSCC and is closely related to its EMT.

### Knockdown of PKD3 significantly inhibits the growth, migration and invasion of OSCC cells

**Fig. 2:**
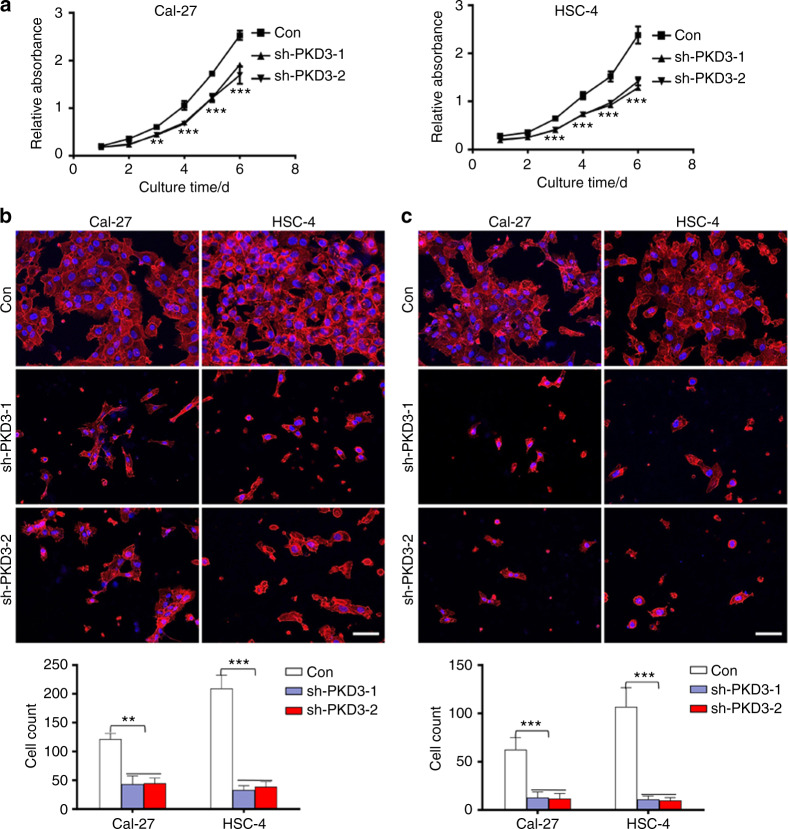
PKD3 downregulation suppresses the proliferation, migration and invasion of OSCC cells. **a** Effects of PKD3 on the proliferation of Cal-27 and HSC-4 cells as evaluated using the CCK-8 assay. **b** Transwell migration assay performed with Cal-27 and HSC-4 cells transfected with shRNA-PKD3 or shRNA-Con plasmids. The number of cells that had migrated per field is shown in the bar graphs; scale bar size = 50 µm. **c** Transwell invasion assay for the indicated cell lines. The number of cells passing through the Matrigel per field is shown in the bar graphs; scale bar size = 50 µm. Nuclei were stained with DAPI (blue). Red represents the cytoskeleton labelled with 555 phalloidin. Data are presented as the means ± SD (*n* = 3). **P < 0.01; ***P < 0.001. PKD3, protein kinase D3; CCK-8, Cell Counting Kit-8; OSCC, oral squamous cell carcinoma

To determine the role of PKD3 in EMT of OSCC, we silenced the expression of PKD3 in the OSCC cell lines Cal-27 (without obvious mesenchymal characteristics) and HSC-4 (with obvious mesenchymal characteristics), the results of which are shown in Fig. 1a. We employed sh-PKD3-1 and sh-PKD3-2 because they were previously determined to effectively knockdown PKD3. First, we analysed the effect of PKD3 on cell proliferation using colony formation and CCK-8 assays. The CCK-8 assay showed that the growth of PKD3-knockdown cells was much slower than that of control cells. Additionally, statistical analysis showed significant differences from the third day after knockdown (Fig. 2a). After 2 weeks, the colony formation rate of PKD3-knockdown cells was significantly reduced (Fig. S2a). Next, we evaluated the effect of PKD3 on the invasion and metastasis of OSCC cells. Transwell migration assays showed that PKD3-knockdown cells migrated more slowly than did control cells (Fig. 2b). PKD3 silencing also significantly reduced the invasive ability of OSCC cells through a Matrigel-coated membrane (Fig. 2c). Furthermore, we examined the effects of PKD3 knockdown on the expression of epithelial and mesenchymal markers using western blotting. As shown in Fig. S2b, the expression of E-cadherin was increased, while that of mesenchymal markers and PD-L1 was decreased in PKD3-knockdown cells. Together, these results indicate that knockdown of PKD3 inhibits the migration, invasion and EMT phenotype of OSCC cells.

### Overexpression of PKD3 promotes the growth, migration and invasion of OSCC cells

**Fig. 3:**
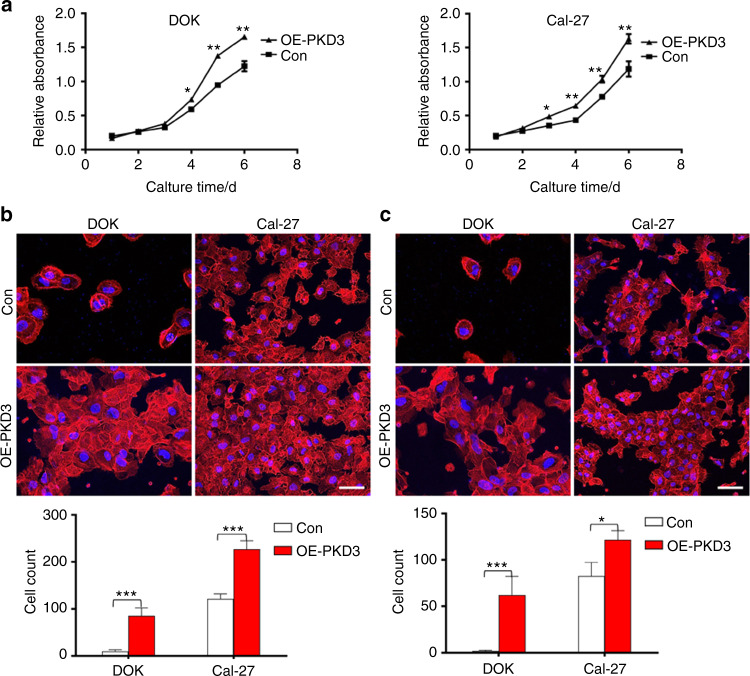
PKD3 overexpression can promote the proliferation, migration and invasion of OSCC cells. **a** Growth curves. **b** Transwell migration assay with DOKs and Cal-27 cells transfected with pCDNA3.1/NT-PKD3 (OE-PKD3) or control plasmid (Con). The number of migrated cells per field is shown in the bar graphs; scale bar size = 50 µm. **c** Transwell invasion assay for the indicated cell lines. The number of invading cells per field is shown in the bar graphs; scale bar size = 50 µm. Nuclei were stained with DAPI (blue). Red represents the cytoskeleton labelled with 555 phalloidin. Data are presented as the means ± SD (*n* = 3). *P < 0.05; **P < 0.01; ***P < 0.001. PKD3, protein kinase D3; OSCC, oral squamous cell carcinoma

According to the results shown in Fig. 1a, we selected two cell lines with the lowest PKD3 expression, namely, DOKs and Cal-27 cells. After screening, we obtained cells stably overexpressing PKD3 and evaluated their proliferation, invasion and migration. Growth curve analysis showed that PKD3-overexpressing cells also proliferated much faster than did control cells. DOKs started showing significant differences from the 4th day, while Cal-27 cells started showing significant differences from the 3rd day (Fig. 3a). We found that the number of clones formed by PKD3-overexpressing cells increased significantly (Fig. S2c). Transwell assays showed that PKD3 overexpression significantly promoted the migration and invasion of DOKs and Cal-27 cells (Fig. 3b, c). Western blot analysis revealed that in PKD3-overexpressing cells, the expression of PD-L1 slightly increased, whereas the expression of epithelial and mesenchymal markers decreased and increased, respectively (Fig. S2d). Collectively, these data demonstrate that PKD3 contributes to regulating EMT of OSCC and that its overexpression promotes the EMT phenotype and metastasis of OSCC.

### PKD3 plays an important role in the EMT of OSCC induced by the PD-1/PD-L1 pathway

**Fig. 4:**
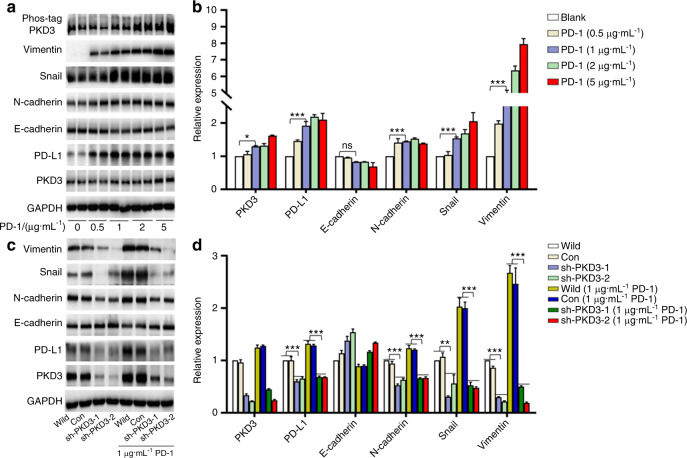
The role of PKD3 in the EMT phenotype of OSCC induced by the PD-1/PD-L1 pathway. **a, b** Western blotting was used to detect the levels of PKD3, PD-L1 and EMT markers in Cal-27 cells treated with the PD-1 fusion protein. **c, d** Protein levels of PKD3, PD-L1 and EMT markers after stable knockdown of PKD3 in Cal-27 cells cultured in the presence or absence of PD-1 fusion protein. Data are presented as the means ± SD (*n* = 3). *P < 0.05; **P < 0.01; ***P < 0.001; ns, nonsignificant; PKD3, protein kinase D3; OSCC, oral squamous cell carcinoma; PD-1, programmed cell death protein 1; PD-L1, programmed death ligand-1; EMT, epithelial–mesenchymal transition

Programmed death ligand-1 (PD-L1) can interact with programmed cell death protein 1 (PD-1) expressed on activated B cells, T cells and natural killer (NK) cells, thus activating the PD-1/PD-L1 pathway, leading to T cell apoptosis or loss of function and downregulating the anti-tumour response of T cells.^26^ However, how activation of the PD-1/PD-L1 pathway affects tumour cells is largely unknown. To answer this question, we stimulated Cal-27 cells with different concentrations of the PD-1 fusion protein for 24 h. As shown in Fig. 4a, b, E-cadherin expression was slightly lower while PKD3 and PD-L1 expression was significantly higher in cells treated with 1, 2 and 5 μg·mL^−1^ PD-1 fusion protein than those in cells treated with a low concentration (0.5 μg·mL^−1^) of PD-1 or untreated cells. We then examined the activation of PKD3 using Phos-tag SDS-PAGE immunoblots. PKD3 phosphorylation began to significantly increase after treatment with 1 μg·mL^−1^ PD-1. Intriguingly, stimulation of cells with all four tested concentrations of PD-1 significantly upregulated the expression of N-cadherin, Snail and Vimentin. Moreover, the levels of Snail and Vimentin gradually increased with increasing concentrations of the PD-1 fusion protein. These data suggest that the binding of the PD-1 fusion protein to PD-L1 promotes EMT in OSCC.

Subsequently, we investigated whether PD-1/PD-L1 pathway-induced EMT in OSCC was dependent on PKD3. For this purpose, we examined the expression of epithelial and mesenchymal markers after 24 h of culture in the presence or absence of 1 μg·mL^−1^ PD-1 fusion protein. The expression of PD-L1, N-cadherin, Snail and Vimentin was increased after treatment with PD-1; however, knockdown of PKD3 significantly reduced the expression of PD-L1 and mesenchymal markers but increased the expression of E-cadherin (Fig. 4c, d). Taken together, these results show that knockdown of PKD3 can effectively block EMT induced by the PD-1/PD-L1 pathway.

### PKD3 regulates EMT and PD-L1 through ERK1/2 and STAT1/3 signalling, establishing a positive feedback loop with PD-L1

**Fig. 5:**
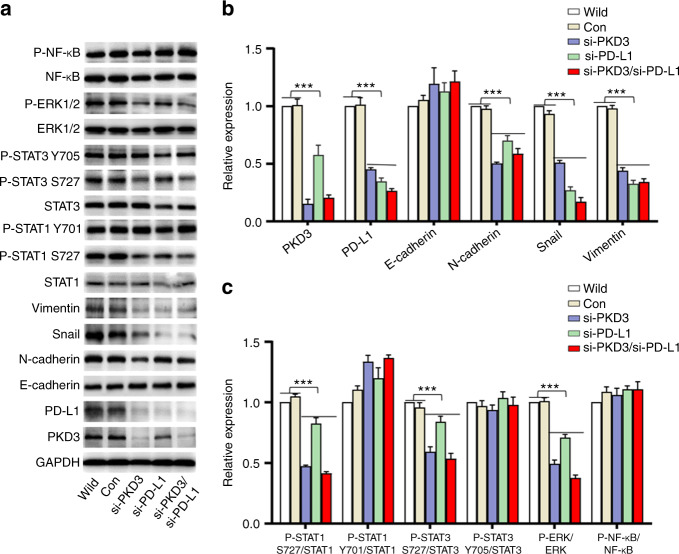
Both PKD3 and PD-L1 activate the ERK1/2 and STAT1/3 pathways to promote EMT in OSCC cells. **a** Activation of EMT-related factors and the expression of EMT markers in Cal-27 cells transfected with PKD3 and/or PD-L1 siRNAs were analysed by western blot. **b** Quantification of PKD3, PD-L1 and EMT markers by densitometry analysis. **c** Densitometric analysis of changes in the abundance of phosphorylated substrates and total proteins normalised to the expression of GAPDH as a loading control. Data are presented as the means ± SD (*n* = 3). ***P < 0.001. PKD3, protein kinase D3; OSCC, oral squamous cell carcinoma; ERK, extracellular signal-regulated kinase; STAT, signal transducer and activator of transcription; NF-κB, nuclear factor-kappa B; PD-L1, programmed death ligand-1; EMT, epithelial-mesenchymal transition

To further explore the mechanism of PKD3-induced OSCC migration and tumour growth, we silenced PKD3 and PD-L1 in Cal-27 cells using siRNAs. As shown in Fig. 5a, b, the expression of PD-L1 was significantly reduced in PKD3-knockdown cells. In these cells, the levels of EMT markers showed the same changes previously observed in sh-PKD3-transfected cells. Additionally, knockdown of PKD3 significantly inhibited the phosphorylation of STAT1/3 at Ser727 but not the phosphorylation of tyrosine residues. Phosphorylation of ERK1/2 was also significantly reduced after PKD3 knockdown. Considering that PKD3 could regulate the expression of PD-L1, which is closely associated with EMT, we speculated that the regulation of EMT by PD-L1 might also involve the ERK and STAT signalling pathways. Interestingly, knockdown of PD-L1 also reduced PKD3 expression. Following PD-L1 knockdown, the expression of mesenchymal markers was significantly reduced, whereas the expression of E-cadherin was slightly increased. Furthermore, knockdown of PD-L1 significantly reduced the phosphorylation of STAT1/3 at serine residues, as well as the phosphorylation, but not expression, of ERK1/2. Double knockdown of PKD3 and PD-L1 inhibited the phosphorylation of ERK1/2 and STAT1/3 on serine residues more effectively than single knockdown of either gene (Fig. 5c). In addition, double knockdown of PKD3 and PD-L1 significantly inhibited the EMT of OSCC, + similar to the single knockdown of PKD3. However, knockdown of neither PKD3 nor PD-L1 could significantly alter NF-κB pathway activity.

**Fig. 6:**
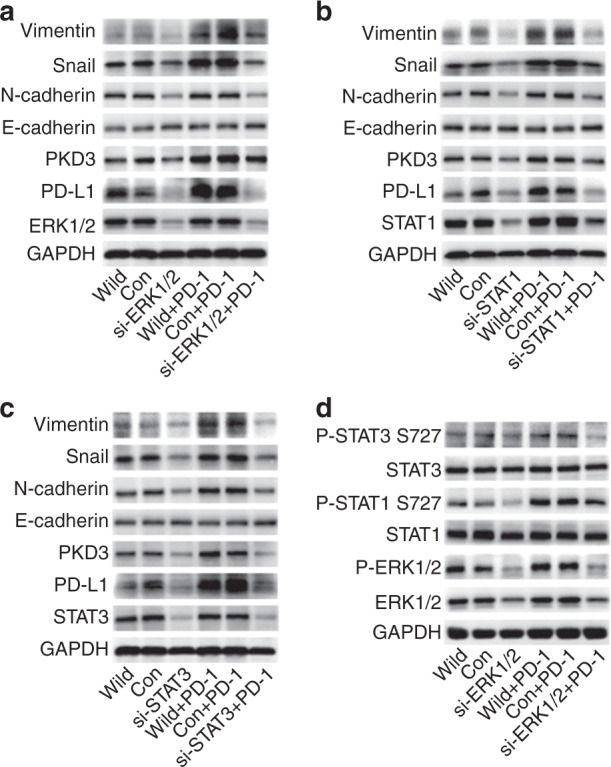
The ERK1/2 and STAT1/3 pathways modulate PKD3 and PD-L1 expression and regulate EMT in OSCC cells. **a**–**c** PKD3, PD-L1, and EMT were detected by western blot after transfection of ERK1/2, STAT1, and STAT3 siRNAs into Cal-27 cells cultured in the presence or absence of PD-1 fusion protein. **d** Western blot analysis of ERK1/2 phosphorylation and STAT1/3 phosphorylation at S727 after transfection of ERK1/2 siRNA in Cal-27 cells cultured in the presence or absence of PD-1 fusion protein. PKD3 protein kinase D3, ERK extracellular signal-regulated kinase, STAT signal transducer and activator of transcription, PD-L1 programmed death ligand-1, EMT epithelial–mesenchymal transition, OSCC oral squamous cell carcinoma, PD-1 programmed cell death protein 1

As ERK1/2 and STAT1/3 play a crucial role in EMT, we analysed their effect on EMT of OSCC cells. Following knockdown of ERK1/2 and STAT1/3, the expression of PKD3 and PD-L1 was effectively suppressed in Cal-27 cells. Moreover, after silencing ERK1/2 and STAT1/3, the expression of mesenchymal markers in OSCC cell lines was still significantly inhibited, even upon stimulation with PD-1 fusion protein, whereas E-cadherin expression was only slightly affected (Fig. 6a–c). Furthermore, the PD-1/PD-L1 interaction significantly increased the phosphorylation of ERK1/2 and STAT1/3; however, the phosphorylation of STAT1/3 at Ser727 was significantly inhibited after ERK1/2 knockdown (Fig. 6d). In conclusion, PKD3 and PD-L1 regulate the phosphorylation levels of ERK1/2 and STAT1/3. Interestingly, ERK1/2 and STAT1/3 in turn can also affect the expression of PKD3 and PD-L1 and thereby regulate EMT in OSCC. These data indicate that PKD3 regulates PD-L1 expression and EMT in OSCC through ERK1/2 and STAT1/3, suggesting a positive feedback mechanism between them.

### PKD3 promotes the growth and lymph node metastasis of OSCC in vivo

**Fig. 7:**
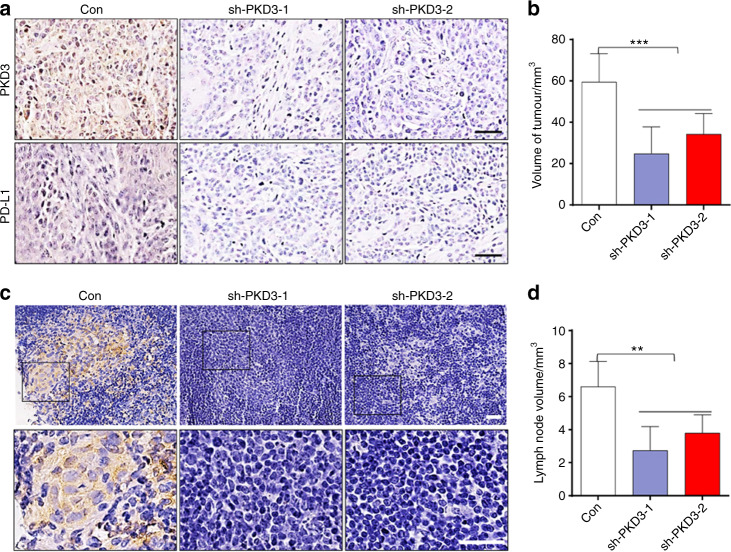
PKD3 promotes the growth and lymph node metastasis of OSCC cells in vivo. **a** Immunohistochemical staining of PKD3 and PD-L1 in subcutaneous footpad tumours. Black scale bars are 50 m. **b** Nude mouse footpads were inoculated with Cal-27 Con, sh-PKD3-1 and sh-PKD3-2 cells. Four weeks later, subcutaneous tumours of the footpads were collected, and their volumes were measured. **c** Immunohistochemical staining of GFP was used to evaluate the ratio of metastatic lymph nodes among different groups; scale bar size = 30 m. **d** Popliteal lymph nodes volumes. Data are presented as the means SD (*n* = 9 per group). **P < 0.01; ***P < 0.001. PKD3, protein kinase D3; PD-L1, programmed death ligand-1; OSCC, oral squamous cell carcinoma; LNs, lymphnodes; GFP, green fluorescent protein

To study the effects of PKD3 on the growth and lymph node metastasis of OSCC in vivo, we injected Cal-27-sh-Con, Cal-27-sh-PKD3-1, or Cal-27-sh-PKD3-2 cells into the footpads of mice to establish a xenograft model. After 4 weeks, the mice were sacrificed, the size of the footpad tumours was measured, and the popliteal lymph nodes were removed and measured. IHC staining revealed that the expression of PD-L1 decreased with knockdown of PKD3 (Fig. 7a). Moreover, tumour size and lymph node metastasis were most pronounced in the Cal-27-sh-Con group and were smaller in the Cal-27-sh-PKD3-1 and Cal-27-sh-PKD3-2 groups (Fig. 7b, d). Since the lentiviral plasmids were labelled with green fluorescent protein (GFP), transfected cancer cells that metastasised into lymph nodes could be identified by IHC staining of GFP (Fig. 7c). Subsequently, we calculated the lymph node metastasis rates in different groups. This rate was highest in the Cal-27-sh-Con group (78%), followed by the Cal-27-sh-PKD3-1 (11%) and Cal-27-sh-PKD3-2 (22%) groups (Table 1). Importantly, the observed difference in metastasis between the control group and the PKD3-knockdown groups was significant, as determined by Fisher’s exact test (*P* = 0.004). Our experiment thus proved that PKD3 can promote the growth and metastasis of OSCC in vivo.

**Table 1:** Ratio of metastatic nodes among retrieved popliteal lymph

Groups	No. total LNs	No. metastasis LNs	Metastatic ratio/%
Con	9	7	78
sh-PKD3-1	9	1	11
sh-PKD3-2	9	2	22

## Discussion

OSCC is a common malignant tumour of the head and neck characterised by rapid growth and a high metastasis rate. Increasing evidence suggests that EMT is crucial for tumour progression and metastasis. In the initial stages of metastasis, epithelial tumour cells undergo EMT, leading to loss of intercellular adhesions, enhanced mobility and invasion of surrounding tissues.^5,27^ Currently, the treatment of patients with metastatic OSCC remains a major clinical challenge. Therefore, it is urgent to explore the potential mechanism of growth and metastasis of OSCC to determine novel and more effective therapeutic targets.

In this study, we investigated the relevance of PKD3, a poorly understood member of the PKD family, to OSCC. Our research revealed that PKD3 regulates EMT and PD-L1 expression in OSCC through the ERK1/2 and STAT1/3 pathways and establishes a positive feedback loop with PD-L1 to promote tumour growth and metastasis. These findings indicate that PKD3 plays a pivotal role in the progression of OSCC, suggesting PKDs as potential therapeutic targets for the treatment of this type of cancer.

Based on data from the UALCAN database, UCSC Xena, and western blot, PKD3 and PD-L1 are significantly upregulated in OSCC. Previous studies have shown that PD-L1 regulates EMT and promotes tumour growth and metastasis.^23,25,28–32^ Moreover, PD-L1 expression can be modulated by PKD3.^14^ Although this evidence implies that PKD3 may be closely related to EMT, no molecular mechanism has previously been reported between the two. Here, we found that PKD3 was positively correlated with mesenchymal markers and negatively correlated with epithelial markers. Downregulation of PKD3 inhibited the invasion, migration and proliferation of OSCC in vitro; reduced tumour growth and metastasis in vivo; and decreased the expression of mesenchymal markers while increasing that of E-cadherin. PKD3 overexpression instead significantly enhanced the ability of OSCC cells to invade, migrate and proliferate; promoted the expression of mesenchymal markers; and inhibited the expression of E-cadherin. IHC and TCGA analysis showed that the expression of PKD3, the frequency and intensity of PDK3 nuclear staining, and the expression of Vimentin gradually increased with advanced tumour grade. These findings support our view that PKD3 can regulate EMT in OSCC and promote tumour growth and metastasis.

PD-L1 interacts with PD-1 expressed by activated T cells, B cells, NK cells, some dendritic cells (DCs) and tumour-associated macrophages, activating the PD-1/PD-L1 pathway. In turn, activation of this pathway can inhibit the anti-tumour function of the same immune cells, thereby reducing anti-tumour immunity.^26,33–36^ However, the effect of the PD-1/PD-L1 interaction on tumour cells has rarely been reported. Here, we found that the expression of PKD3 and PD-L1 significantly increased after treatment of OSCC cell lines with the PD-1 fusion protein; in parallel, the expression of mesenchymal markers increased, whereas that of epithelial markers decreased, suggesting that the PD-1/PD-L1 pathway can induce EMT in tumour cells. EMT is key to tumour cell remodelling for metastasis.^5^ The interaction between tumour cells and tumour-infiltrating lymphocytes induces the expression of PD-L1 in tumour cells through the STAT pathway; in HNSCC, the expression of PD-L1 is closely related to EMT.^25,37,38^ These results are consistent with ours, which indicate that, in vivo, PD-1-expressing T cells, B cells, and NK cells interact with OSCC cells, promoting EMT and immune escape. This may also be the cause of early lymph node metastasis in OSCC.

**Fig. 8:**
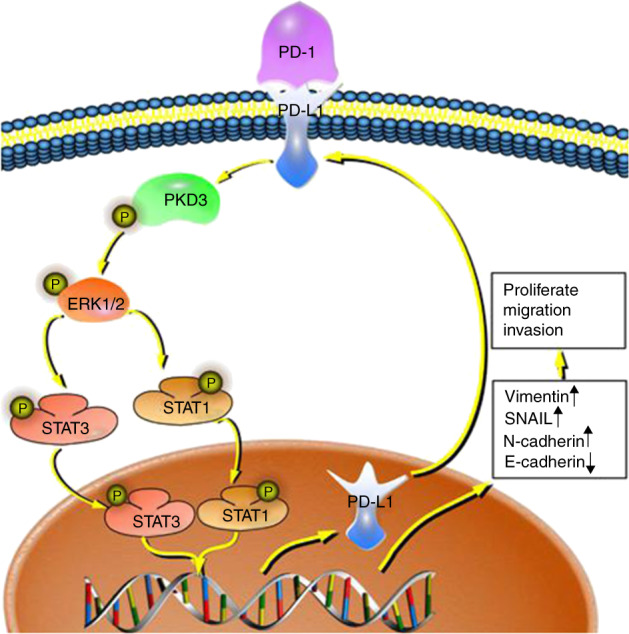
The prediction model of the PKD3/PD-L1 positive feedback loop. PKD3/PD-L1 drives EMT through ERK/STAT1/3 signalling, thus promoting tumour growth and metastasis. PKD3, protein kinase D3; ERK, extracellular signal-regulated kinase; STAT, signal transducer and activator of transcription; PD-L1, programmed death ligand-1; PD-1, programmed cell death protein 1

The nuclear localisation and catalytic activation of PKD3 are linked to the activation of G protein-coupled receptor signalling pathways,^39^ which include the STAT1/3, ERK and NF-κB pathways.^40–43^ These pathways, which are also regulated by PKD3, influence cell growth, metastasis, EMT and other biological processes as well as modulate the expression of PD-L1.^12–14,17–22,24^ The synchronicity of these different biological processes suggests the possibility of a common genetic driver. In OSCC cells, silencing PKD3 can effectively block EMT induced by the PD-1/PD-L1 interaction. Transient knockdown of ERK1/2, STAT1 or STAT3 also abolishes PD-1/PD-L1-induced EMT. As PKD3 mainly induces the activation of ERK1/2 and STAT1/3, we concluded that PKD3 may regulate EMT in OSCC cells through ERK1/2 and STAT1/3. Interestingly, we found that knockdown of PKD3 reduced the expression of PD-L1; conversely, the expression of PKD3 decreased after knocking down PD-L1. Moreover, the PD-1 fusion protein induced the expression of PD-L1 and PKD3. Knockdown of PD-L1 could also affect the activation of ERK1/2 and STAT1/3. PD-L1 was previously shown to regulate the activation of ERK1/2 by binding with RAS, which consequently affects EMT of tumour cells.^32^ Additionally, the activation of STAT1/3 can be mediated by ERK1/2.^44–50^ These findings are consistent with our results, which suggest that there is a positive feedback loop between PKD3 and PD-L1 that drives EMT via ERK/STAT1/3 signalling, ultimately promoting tumour growth and metastasis (Fig. 8).

## Materials and methods

### Cell culture

Human normal oral epithelial keratinocytes (HOKs) were cultured in keratinocyte serum-free medium (KSFM) with bovine pituitary extract (50 μg·mL^−1^) and recombinant human epidermal growth factor (5 ng·mL^−1^) (Gibco, USA). SCC25 cells were cultured in DMEM/F12 medium (Gibco, USA) containing 10% foetal bovine serum (FBS; PAN, Germany), 400 ng·mL^−1^ hydrocortisone, 100 IU·mL^−1^ penicillin, and 100 μg·mL^−1^ streptomycin (HyClone, USA). Dysplastic oral keratinocytes (DOKs) and three other oral squamous cell lines (Cal-27, HSC-4, and HSC-3) were cultured in high-glucose DMEM containing 10% FBS, 100 IU·mL^−1^ penicillin, and 100 μg·mL^−1^ streptomycin. The culture medium for DOKs also contained 5 μg·mL^−1^ hydrocortisone. HOKs were purchased from ScienCell Research Laboratories, Inc. DOKs were supplied by the European Collection of Authenticated Cell Cultures (ECACC). Four OSCC cell lines (Cal-27, HSC-4, SCC25 and HSC-3) were obtained from the American Type Culture Collection (ATCC). All cells were maintained at 37 °C in a humidified atmosphere with 5% CO_2_.

### Plasmid and siRNA transfection

CSHCTR001-LVRU6GP-control and HSH006319-LVRU6GP-PKD3 (clone ID NM_005813.3) shRNA plasmids were purchased from GeneCopoeia. The target sequences are listed in Supplementary Table S1. Specific siRNAs against STAT1, STAT3, ERK1 and ERK2 as well as si-control were synthesised by GenePharma (Shanghai, China; Supplementary Table S2). Specific siRNAs against PKD3 and PD-L1 were purchased from Invitrogen. Lipofectamine 2000 reagent was used to transfect Cal-27 and HSC-4 cells with shRNA plasmids and Cal-27 cells with siRNA according to the manufacturer’s protocol. Puromycin (0.2 μg·mL^−1^) was used to screen Cal-27 and HSC-4 cell lines with stable PKD3 knockdown. DOKs and Cal-27 cell lines stably overexpressing PKD3 were generated similarly. Human PKD3-overexpressing DOKs and Cal-27 cells were established using pEX-U1233-M02 (control: pEX-EGFP-M02; GeneCopoeia).

### Patients and clinical samples

In this study, a total of 34 patients with OSCC who did not receive radiotherapy or chemotherapy before surgery were enroled, and 26 normal oral epithelial tissues were collected from the West China Hospital of Stomatology, Sichuan University. The patients’ clinical information is shown in Supplementary Table S3. All clinical samples were confirmed by pathological examination. This study was approved by the Ethics Committee of West China Hospital of Stomatology, Sichuan University, and all patients gave informed consent.

### Western blot

Total proteins were extracted for western blot detection according to the method described previously.^14^ Protein concentration was measured using the BCA protein assay kit (Beyotime Institute of Biotechnology, China). Approximately 20 μg of protein was separated by SDS-PAGE through 6% or 10% gels and then transferred to PVDF membranes (Bio-Rad, USA). After the PVDF membrane was blocked in 5% skim milk at room temperature for 1 h, the primary antibody was added and incubated overnight on a shaker at 4 °C. Membranes were washed with TBST and then incubated with horseradish peroxidase (HRP)-conjugated anti-mouse or anti-rabbit secondary antibodies (cat. no. 7076 or 7074; 1:2 000; Cell Signaling Technology, Inc.) for 1–2 h at room temperature. An enhanced chemiluminescence (ECL) substrate kit (Millipore, Inc.) and an automatic gel imaging system (Bio-Rad Laboratories, Inc.) were used to detect the luminescent signal of protein bands. Primary antibodies used for western blotting targeted the following proteins: PKD3 (cat. no. 5655; 1:1 000), PD-L1 (cat. no. 13684; 1:1 000), E-cadherin (cat. no. 3195; 1:1 000), N-cadherin (cat. no. 13116; 1:1 000), Vimentin (cat. no. 5741; 1:1 000), Snail (cat. no. 3879; 1:1 000), ERK1/2 (cat. no. 4696; 1:1 000), and phospho-p44/42 MAPK (Erk1/2) (Thr202/Tyr204) (cat. no. 4370; 1:1 000) from Cell Signaling Technology; and STAT1 (cat. no. ab109320; 1:10 000), phospho-STAT1(S727) (cat. no. ab109461; 1:5 000), phospho-STAT1(Y701) (cat. no. ab29045; 1:1 000), STAT3 (cat. no. ab68153; 1:2 000), phospho-STAT3(S727) (cat. no. ab32143; 1:5 000), phospho-STAT3(Y705) (cat. no. ab76315; 1:10 000), NF-κB P65 (cat. no. ab32536; 1:20 000), phospho-NF-κB P65 (cat. no. ab109458; 1:5 000), and GAPDH (cat. no. ab128915; 1:20 000) from Abcam. Phos-tag SDS-PAGE (Wako, Japan) was performed according to the protocol described previously.^51^ The densitometry of the protein bands was quantified using Gel-Pro 32 software (version 3.1, Media Cybernetics, Bethesda, MD, USA).

### Immunohistochemical staining

All excised OSCC tissues and lymph nodes of mice were fixed with 10% neutral formalin, embedded in paraffin, and cut into 5-μm-thick sections. Immunohistochemical staining was performed as previously described.^14^ The antibodies used for this method included PKD3 (cat. No. ab252982; 1:40) from Abcam and PD-L1 (cat. no. 13684; 1:200), E-cadherin (cat. no. 3195; 1:400), and Vimentin (cat. no. 5741; 1:200) from Cell Signaling Technology.

### Cell Counting Kit-8 assay

After serial dilution of the cell suspension, the cells were seeded at a density of 3 × 10^3^ cells per well in 96-well plates (100 μL per well), and at least three replicate wells were set per group. After 24 h, Cell Counting Kit-8 (CCK-8) was used to detect cell proliferation (DOJINDO, Japan). The test was continued for 6 days; the cells were incubated with CCK-8 solution for 1 h before absorbance measurements. The growth curve was made according to the absorbance at 450 nm.

### Cell invasion and migration assay

Cell invasion assays were performed using 24-well Transwell chambers (8-μm pore size; BD Bioscience, USA). Cells (1 × 10^5^) were seeded in the upper chamber, which was coated with 50 μl of Matrigel (5 mg·mL^−1^ in cold medium, BD Bioscience, USA) and cultured with 1% FBS. Then, 600 μL of high-glucose DMEM supplemented with 10% FBS was added to the lower chamber. After incubation at 37 °C for 24 h, the cells were fixed with 4% paraformaldehyde for 15 min. The cells in the upper chamber were removed, which was then washed with PBS three times, and the invading cells were stained with Acti-stain^TM^ 555 phalloidin (Cytoskeleton, Inc. USA) according to the manufacturer’s protocol. The chamber membrane was placed on a glass slide, and 10 fields of view were randomly selected under the microscope to take pictures and count stained cells. ImageJ 1.48v software was used to count the number of cells. Each group had three replicates.

Cell migration assays were performed similarly to cell invasion assays but without Matrigel in the upper chamber. The same methods described above were used to fix, stain, photograph and count the cells.

### Popliteal lymph node metastasis model

The animal experiments were approved by the Ethics Review Committee of the West China Hospital of Stomatology, Sichuan University (permit number: WCHSIRB-D-2017-211). Female BALB/c-nu mice (4–5 weeks old) were purchased from the Transgenic Engineering Mouse Centre of the West China Hospital of Sichuan University. The mice were randomly divided into three groups (Con, sh-PKD3-1 and sh-PKD3-2), with nine mice per group. Cal-27 cells (4 × 10^6^ per mouse) were injected subcutaneously into the footpad of the left hind leg. All transfected cells stably expressed GFP. Four weeks later, the nude mice were euthanized. Primary tumours and popliteal lymph nodes were removed. The tissues were fixed with 10% neutral formalin and paraffin embedded. Serial 5-μm-thick slices were cut and analysed by immunohistochemistry (IHC) with rabbit anti-human PKD3 polyclonal antibody (cat. no. ab252982; 1:40; Abcam), rabbit anti-human PD-L1 polyclonal antibody (cat. no. 13684; 1:200; Cell Signaling Technology) or rabbit anti-GFP monoclonal antibody (cat. no. ab183734; 1:200; Abcam).

### Statistical analysis

Correlation analysis was performed using Origin 2018 software (Origin Lab). Other analyses were performed using GraphPad Prism software (version 6; GraphPad Software, Inc.) using one-way ANOVA followed by Tukey’s multiple comparison test. Fisher’s exact test was used to analyse the relationship between PKD3 expression and lymph node metastasis. *P* < 0.05 was considered statistically significant.

## Conclusion

In conclusion, we determined that the PD-1/PD-L1 interaction could promote EMT in OSCC. Furthermore, we demonstrated that PKD3 regulates PD-L1 expression through the ERK/STAT1/3 pathway and that there is a positive feedback loop between PKD3 and PD-L1 related to driving EMT and promoting the growth and metastasis of OSCC. Our study thus suggests that targeting PKD3 could be an effective strategy to block EMT, providing a new potential avenue for the treatment of OSCC.

## Supplementary information

Supplementary Table S1

Supplementary Table S2

Supplementary Table S3

Figure S1

Figure S2
