# Supplementary material for: PKD3 promotes metastasis and growth of oral squamous cell carcinoma through positive feedback regulation with PD-L1 and activation of ERK-STAT1/3-EMT signalling
Source: Int J Oral Sci. 2021 Mar 10;13:8. doi: 10.1038/s41368-021-00112-w (PMC7946959; doi:10.1038/s41368-021-00112-w)
Supplement: Supplementary file 1 — Supplementary Table S1 [file 41368_2021_112_MOESM1_ESM.docx]

Table S1 ShRNA target sequences used in this study.

| shRNA | Target Sequence |
| --- | --- |
| sh-PKD3-1 | ccagattcgtccacatactct |
| sh-PKD3-2 | gctcctactttctgtgattac |
