# Supplementary material for: PKD3 promotes metastasis and growth of oral squamous cell carcinoma through positive feedback regulation with PD-L1 and activation of ERK-STAT1/3-EMT signalling
Source: Int J Oral Sci. 2021 Mar 10;13:8. doi: 10.1038/s41368-021-00112-w (PMC7946959; doi:10.1038/s41368-021-00112-w)
Supplement: Supplementary file 2 — Supplementary Table S2 [file 41368_2021_112_MOESM2_ESM.docx]

Table S2 SiRNA sequences used in this study.

| siRNA | sequences |
| --- | --- |
| si-STAT1 | 5'-CAC GAG ACC AAU GGU GUG GdTdT-3' |
| si-STAT3 | 5'- AAC AUC UGC CUA GAU CGG CUAdTdT-3' |
| si-ERK1 | 5'-GAC CGG AUA ACC UUU AdTdT-3' |
| si-ERK2 | 5'-CACCACCUGUGAUCUCAAGUUdTdT-3' |
| si-Control | 5'- UUCUCC GAA CGU GUC ACG UdTdT-3' |
