# Supplementary material for: PKD3 promotes metastasis and growth of oral squamous cell carcinoma through positive feedback regulation with PD-L1 and activation of ERK-STAT1/3-EMT signalling
Source: Int J Oral Sci. 2021 Mar 10;13:8. doi: 10.1038/s41368-021-00112-w (PMC7946959; doi:10.1038/s41368-021-00112-w)
Supplement: Supplementary file 3 — Supplementary Table S3 [file 41368_2021_112_MOESM3_ESM.docx]

Table S3 Clinical characteristics of 34 OSCC patients.

| Characteristics (34) | Case | % |
| --- | --- | --- |
| Age (years) |  |  |
| <57 | 16 | 47.1% |
| >57 | 18 | 52.9% |
| Sex |  |  |
| Male | 26 | 76.5% |
| Female | 8 | 23.5% |
| TNM stage |  |  |
| I-Ⅱ | 15 | 44.1% |
| III‑IV | 19 | 55.9% |
| Histological grade |  |  |
| Grade 1 | 12 | 35.3% |
| Grade 2 | 18 | 52.9% |
| Grade 3 | 4 | 11.8% |
| OSCC, oral squamous cell carcinoma; TNM, Tumor‑Node‑Metastasis. | | |
