# Supplementary figures and images for: PKD3 promotes metastasis and growth of oral squamous cell carcinoma through positive feedback regulation with PD-L1 and activation of ERK-STAT1/3-EMT signalling
Source: Int J Oral Sci. 2021 Mar 10;13:8. doi: 10.1038/s41368-021-00112-w (PMC7946959; doi:10.1038/s41368-021-00112-w)

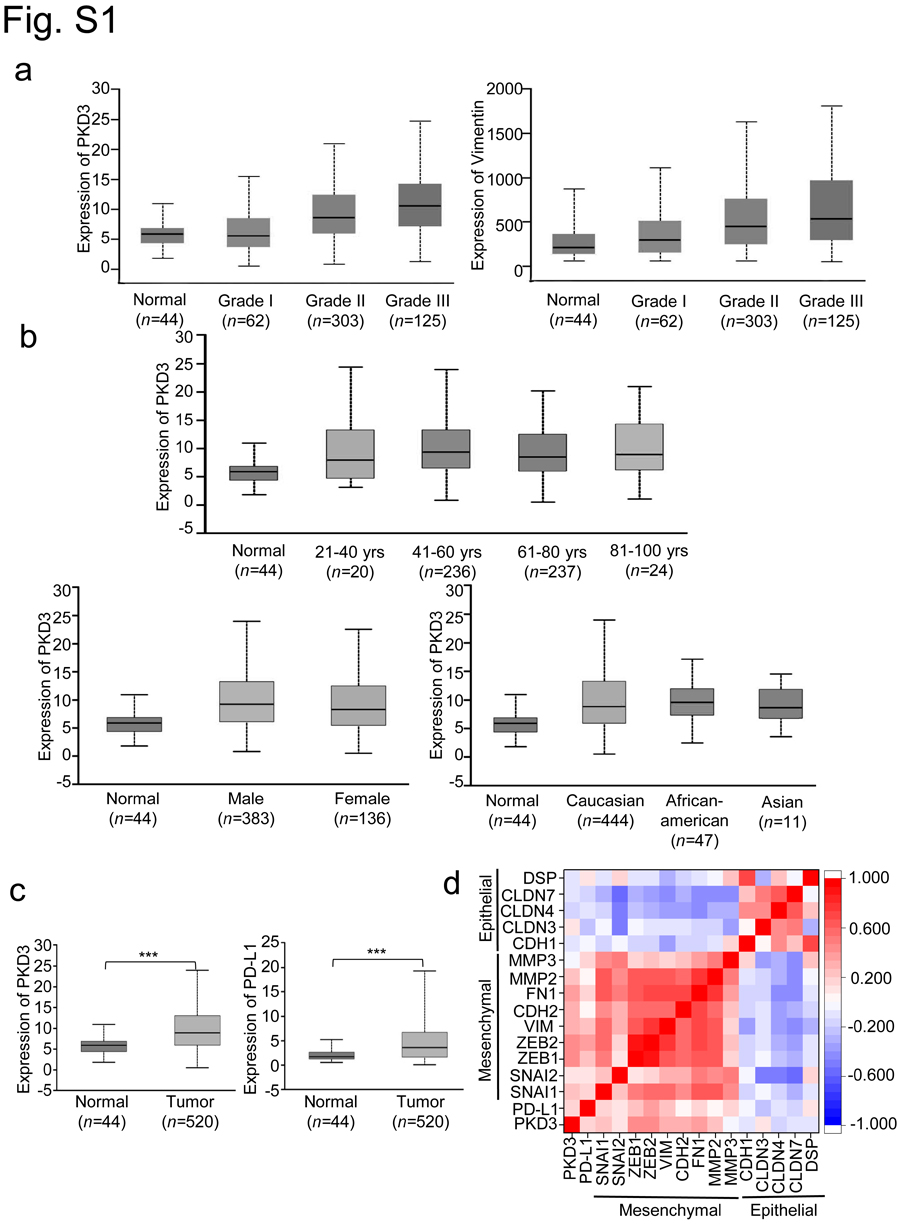

Supplement: Supplementary file 4 — Figure S1 [file 41368_2021_112_MOESM4_ESM.jpg]

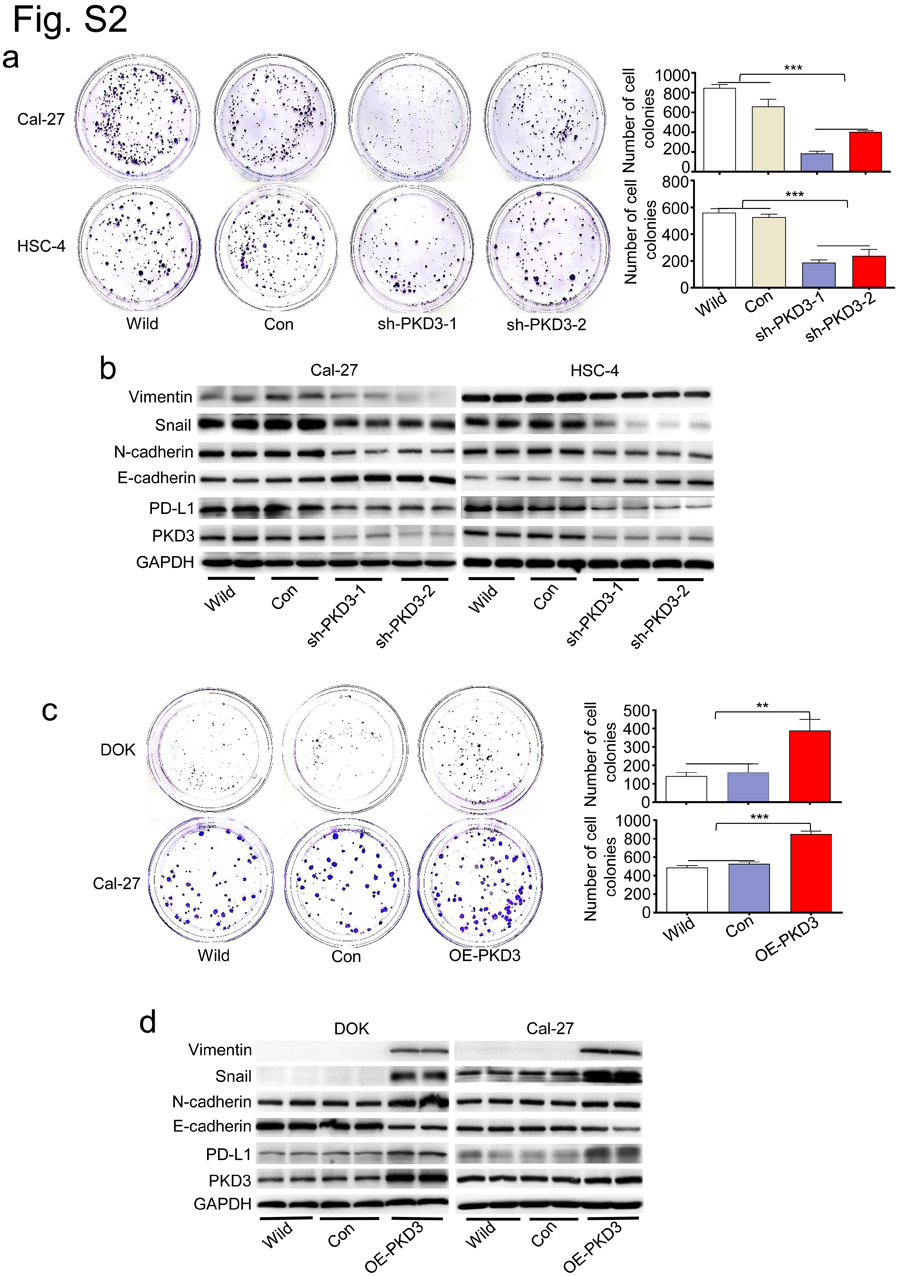

Supplement: Supplementary file 5 — Figure S2 [file 41368_2021_112_MOESM5_ESM.jpg]
